# Impact of Solvent Evaporation and Curing Protocol on Degree of Conversion of Etch-and-Rinse and Multimode Adhesives Systems

**DOI:** 10.1155/2019/5496784

**Published:** 2019-04-11

**Authors:** Ceci Nunes Carvalho, Marcos Daniel Septímio Lanza, Letícia Gomes Dourado, Edilausson Moreno Carvalho, José Bauer

**Affiliations:** ^1^School of Dentistry, CEUMA University, Rua Josué Montello No 1, Renascença II, 65075120 São Luís, MA, Brazil; ^2^Department of Restorative Dentistry, College of Dentistry, Federal University of Minas Gerais, Presidente Antonio Carlos Av 6627, Pampulha, Belo Horizonte, MG, Brazil; ^3^Discipline of Dental Materials, School of Dentistry, University Federal of Maranhão (UFMA), Av. dos Portugueses, 1966, 65085680 São Luis, MA, Brazil

## Abstract

**Objectives:**

This study evaluated the effect of air-drying time and light-curing time on the degree of conversion (DC) of three etch-and-rinse adhesive systems: ONE-STEP (OS) and ONE-STEP plus (OSP), Ambar (AMB), and two multimode adhesive systems: All-Bond Universal (ABU) and ScotchBond Universal (SBU) by Fourier transform infrared (FTIR) analysis.

**Materials and Methods:**

The DC of each adhesive system was analyzed with six experimental different protocols: (1) immediate light curing for 10 s without solvent volatilization; (2) 10 s solvent volatilization with air stream plus 10 s light curing; (3) 60 s solvent volatilization with air stream plus 10 s light curing; (4) immediate light curing for 20 s without solvent volatilization; (5) 10 s solvent volatilization with air stream plus 20 s light curing; and (6) 60 s solvent volatilization with air stream plus 20 s light curing. FTIR spectra were obtained, and the DC was calculated by comparing the ratio of aliphatic/aromatic double carbon bonds before and after light activation (Bluephase 20i). The DC means were analyzed by three-way analysis of variance (ANOVA) and post hoc Tukey tests (*α* = 0.05).

**Results:**

Three-way ANOVA showed statistically significant adhesive, air-drying, and light-cured time (*p* < 0.001). In general, there was a trend of increased DC when the adhesives were dried and cured for longer times, but that was not observed for all the adhesives tested. The acetone-based adhesive systems require an air-drying prior to light activation. The light-curing time of 20 s increases the DC of all materials tested.

**Conclusion:**

The results suggested that the DC of the adhesive systems tested was material dependent. In general, the protocol with solvent evaporation for 10 seconds with air syringe plus 20 seconds of light curing finds the high values of DC.

## 1. Introduction

The development of adhesive systems has completely changed the traditional concepts of dentistry. Besides the complexity and number of steps involved with those application, researchers and manufacturers have aimed to simplify the clinical procedure by reducing the number of bonding steps and, thus, working time [1]. Most simple-to-use one-step adhesives contain a combination of hydrophilic and hydrophobic monomers, diluents, and photoinitiator systems, all provided in a single bottle solution containing ethanol or acetone as solvents [2, 3].

The solvent has an important role on adhesive infiltration into the wet dentin substrate. The monomer interdiffusion has been demonstrated as the fundamental mechanism in achieving effective dentine bonding [4]. Therefore, the solvents should be completely removed during clinical application of the adhesive with an air-drying [5–7]. Otherwise, the remaining solvent in the adhesive may jeopardize polymerization due to the dilution of monomers and may result in voids and increase the permeability of the cured adhesive layer [8, 9]. This might have an adverse effect on the performance of the resin-dentin bonds [6]. In the same way, the high solvent concentration within the adhesive layer prior to polymerization prevents the attainment of a high cross-linking polymer [10] and leads to pores between interfacial layers [11].

Clinicians should attempt to remove the highest amount of solvent to achieve an adequate monomer conversion [12]. It was previously demonstrated that extending the photoactivation time of simplified adhesives beyond those recommended by the manufacturers resulted in improved polymerization and reduced permeability, and it appeared to be a possible mean for improving the performance of these adhesives [13, 14]. There is no consensus; however, regarding the air-drying and polymerization time for 1-step self-etch or for 2-step etch-and-rinse, none have information about the new universal adhesive systems.

Thus, the aim of this study was to evaluate the DC of five adhesive systems after different solvent evaporations methods and curing time protocols by Fourier transform infrared (FTIR) analysis. The null hypothesis tested was that different protocols of air-drying and curing times would not result in significant differences in the DC of the adhesives tested.

## 2. Materials and Methods

**Figure 1:**
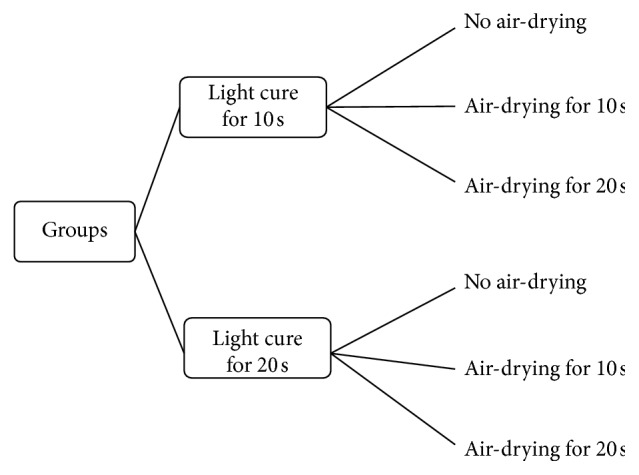
Experimental design.

The materials investigated were three etch-and-rinse adhesive systems: ONE-STEP (Bisco Inc., Schaumburg, IL, USA) and ONE-STEP Plus (Bisco Inc., Schaumburg, IL, USA), Ambar (FGM, Joinville, SC, Brazil), and two multimode adhesive systems: All-Bond Universal (Bisco Inc., Schaumburg, IL, USA) and ScotchBond Universal (3M/ESPE, St. Paul, MN, USA). The chemical compositions, solvents, and manufacturers used in this study are listed in Table 1. Five experimental groups for each adhesive were formed according to different protocols of air-drying and curing times (Figure 1).

Solvent evaporation was performed at 3 bar using a pressure regulator, and the air nozzle was held at 90° to the dentin surface at a distance of 20 cm distance from the sample. Samples were light cured either for 10 s or 20 s, with a LED light-curing unit (Bluephase 20i/Ivoclar Vivadent Inc., Amherst, NY, USA) using the high curing mode, and at 5 mm from the sample surface.

### 2.1. DC Analysis

The DC was analyzed by the FTIR spectrometer (IRPrestige-21, Shimadzu Corporation, Kyoto, Japan) equipped with an attenuated total reflectance crystal (ATR-MIRacle™ Single Reflection Horizontal, Pike Technologies, Inc., Madison, WI, USA).

The absorption spectra of each uncured adhesive were obtained by placing two drops of each adhesive solution directly to the surface of the ATR diamond crystal. The absorption spectra of each cured adhesives specimens were obtained by dispensing two drops of the tested adhesive on an individual acetate strip, and it was subsequently air-dried and light-cured accordingly for each experimental group. After curing, the flat cured surface of the adhesive was firmly placed against the ATR crystal to collect the spectra. FTIR readings were carried out at 22 ± 1°C with 50% relative humidity.

For the adhesive systems containing aromatic vinyl bonds of bisphenol and aliphatic bonds of the methacrylate functional group, the measurement of DC was done by evaluating the performance with the intensity of the spectra of the aromatic component band with the main peak around 1608 cm^−1^, relative to the band with the main absorbance peak of aliphatic carbon-to-carbon double bonds around 1638 cm^−1^, which changes with the polymerization of the composite [12].

**Figure 2:**
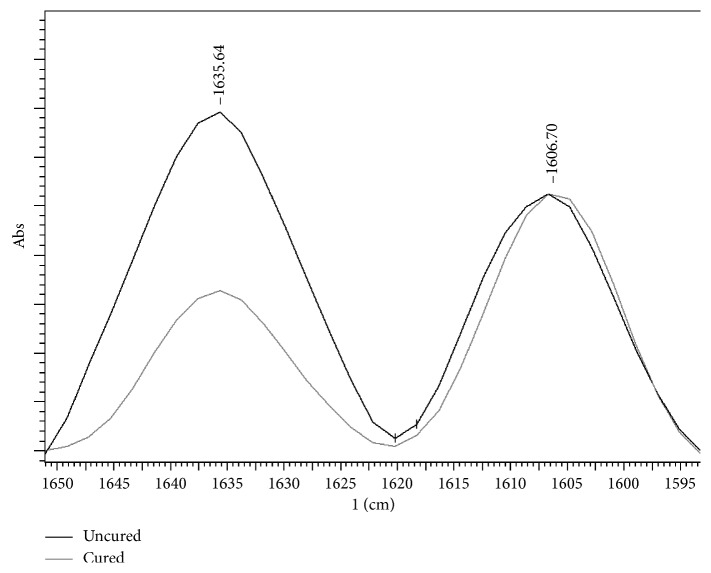
Intensity of the spectra of the Bis-GMA-based adhesive system.

Thus, for the adhesive systems ABU, AMB, OS, OSP, and SBU, the spectra were obtained from the range between 1650 cm^−1^ and 1595 cm^−1^ with 30 scans at 4 cm^−1^ of resolution [14]. The DC (%) was calculated using the following equation: DC (%) = 100 × [1 − (*R*_(cured)_/*R*_(uncured)_)] [15], where *R* represents the ratio between the absorbance peak around 1638 cm^−1^ and 1608 cm^−1^ (Figure 2).

Statistical analysis was performed using the SigmaPlot 13 software (SigmaPlot v. 13.0, Systat Software Inc., San Jose, USA). The normality and equality of variance assumptions were statistically analyzed by the Shapiro–Wilk test and Brown–Forsythe test. A three-way ANOVA (*adhesive vs. air-drying vs. light-cured time*) and Tukey's multiple comparisons test was used to analyze the data at a *α* = 0.05.

## 3. Results

The means and standard deviations of the degree of conversion (%) values are presented in Table 2. Three-way ANOVA analysis showed that there were significant interactions between all three parameters (*F* = 7.65; *p* < 0.001). The increase of light time and air-drying showed significant increases in DC for all tested adhesives (Table 2).

On the contrary, the degree of conversion of AMB and SBU was not influenced by the volatilization technique. In general, there was a trend of increased DC when the adhesives were dried (10–60 s) and cured for longer time (20 s), but that was not observed for all the adhesives tested.

## 4. Discussion

The use of the air-drying has a fundamental role for the evaporation of the solvent in the adhesive systems. The application time, distance, and air pressure are variables that guide the behavior of adhesive systems. In general, when a higher air-drying pressure is used, the longer distance is indicated [16]. In this way, we chose the high constant air pressure (3 bar) and a large air distance for the material (20 cm) and analyze the time of the air-drying.

All adhesive systems used in the present study are simplified, and these materials have a large amount of solvent and hydrophilic monomers. Adhesive systems with large quantities of hydrophilic monomers are characterized by suboptimal polymerization [17] and hence the importance of increasing their degree of conversion with changing air-drying time and curing time. On the contrary, adhesive systems based on solvent-free hydrophobic monomers show a more complete polymerization [13].

Several studies have reported that solvents and water should be eliminated from the dentin surface before light curing with the air-drying procedure [6, 18, 19]. Some studies show that a short adhesive air-drying time (5–10 s) might be insufficient to obtain adequately durable bonding to dentin; instead, air-drying should be performed for longer periods (15–30 s) [20, 21].

However, the results of this study showed that the DC of bonding agents did not depend solely as a function of the air-drying, and it depend also on the light cured time. Most of the adhesives tested in this study showed a high degree of conversion with the increase in the curing time and the air-drying. And, the highest values of DC were found with a curing time of 20 s and air-drying in 10 s. So, the null hypothesis tested in this study was rejected because the degree of conversion of this adhesive was affected by different protocols of air-drying and curing times.

Besides, the increase of the curing time leads to a higher energy dose [22]. Some authors proposed to prolong the curing time beyond the time period recommended by the manufacturers [13, 23]. These studies showed that by extending the curing times, the degree of conversion of adhesive could be improved. The increase of curing time can lead the heat generated from the curing unit also to facilitate solvent volatilization [12] that reduces the distance between the monomers [24] and increases the DC.

Previous study showed that air-drying had a significant effect on the evaporation of primer components, and that the degree of evaporation depends largely on the primer solvents employed [8]. The results showed that air-drying for a long time (10 s–60 s) resulted in significant increases in the DC for two acetone-based adhesives (OS, OSP) regardless of cured time. Acetone has a higher vapor pressure than ethanol and water [25]; for that reason, adhesives containing this acetone increase considerably their DC with increasing curing time and air-drying time.

Bail et al. [18] showed that longer solvent evaporation regimes were the most effective maneuvers in evaporating acetone-based adhesives, and this can optimize the degree of conversion and reduce the solubility/water sorption tendency. Some studies have shown the use of an air-drying to accelerate solvent evaporation [26], and this has an effect on the microtensile bond strength and mechanical properties of the adhesive system [7, 9, 27].

Although all manufacturers indicate the air-drying step after applying bonding agents to evaporate water and solvents from the adhesive solution, some products tested in this study (AMB and SBU) were not influenced by evaporation methods, representing less sensitive materials for clinical use.

 In the present study, different conversion degree values were found for adhesive systems with the same solvent. This may have occurred because of the different organic matrices present in these materials. These matrices may retain different amounts of solvent due to the polarity of the monomers. The resin polarity influences the number of hydrogen bonding sites and the attraction between the polymer and solvent [28]. Another way, ethanol-based adhesive ABU contains 30–60 wt.% ethanol, whereas SBU contains a lower concentration, 10–15 wt.% [29]. The monomer concentration increases dramatically with the evaporation of ethanol, reducing the vapor pressure of the remaining ethanol [30, 31]. This increase in monomer concentration prevents further solvent evaporation, resulting in residual ethanol being trapped inside the adhesive layer [25]. This explains the lower DC observed in our study for ABU, which has a relatively high ethanol content [29].

For this reason, the optimal time of the evaporation solvent must be determined for each adhesive formulation in relation to the resin composition and solvent type. Even though a complete removal of the solvent is impossible, the evaporation should be maximized, and the adhesive layer must be air-dried to ensure adequate solvent evaporation [32].

## 5. Conclusions

The DC of the adhesive systems tested was material dependent. Air-drying for long time (10 s–60 s) is mandatory to acetone-based adhesives. The ethanol/water-based adhesive systems tested benefited from extended light cured time either with or without air application. One ethanol-based adhesive system tested (AMB) was not influenced by solvent evaporation techniques or by light-curing time.

## Figures and Tables

**Table 1:** Composition, solvent, classification, manufacturer, and batch numbers of the adhesive systems used in this study.^*∗*^

Adhesive systems	Composition	Solvent	Classification	Manufacturer	Batch numbers
ONE-STEP (OS)	BPDM, HEMA, Bis-GMA	Acetone	Etch-and-rinse	Bisco Inc., Schaumburg, IL, USA	1200013326
ONE-STEP Plus (OSP)	BPDM, HEMA, Dental Glass	Acetone	Etch-and-rinse	Bisco Inc., Schaumburg, IL, USA	1200013362
Ambar (AMB)	UDMA, HEMA, methacrylate acidic monomers, methacrylate hydrophilic monomers.	Ethanol	Etch-and-rinse	FGM, Joinville, SC, Brazil	41210
ScotchBond Universal (SBU)	MDP phosphate monomer, dimethacrylate resins, HEMA, silane, initiators, fillers	Ethanol/water	Multimode	3M ESPE, St. Paul, MN, USA	497908
All-Bond Universal (ABU)	Bis-GMA, MDP monomers	Ethanol	Multimode	Bisco Inc., Schaumburg, IL, USA	1300001314

HEMA: 2-hydroxyethyl methacrylate; BPDM: biphenyl dimethacrylate; Bis-GMA: bisphenol A diglycidyl ether dimethacrylate; UDMA, urethane dimethacrylate. ^*∗*^Source: manufacturer documentation.

**Table 2:** DC means and standard deviations (%) of adhesive systems according to testing groups.^*∗*^

Adhesive system	Light-curing time for 10 s			Light-curing time for 20 s		
	No air-drying	Air-drying for 10 s	Air-drying for 60 s	No air-drying	Air-drying for 10 s	Air-drying for 60 s
ONE-STEP (OS)	2.0 (0.6)^R^	10.9 (5.5)^P,Q,R^	27.0 (4.8)^L,M^	14.0 (1.2)^O,P,Q^	27.9 (6.9)^L,M^	38.1 (4.1)^J,K^
ONE-STEP Plus (OSP)	15.6 (2.9)^O,P,Q^	17.5 (3.7)^N,O,P^	31.1 (1.1)^K,L^	21.7 (1.3)^M,N,O^	47.5 (3.3)^G,H,I^	47.9 (2.7)^F,G,H,I^
Ambar (AMB)	56.6 (4.1)^B,C,D,E,F^	54.8 (2.0)^C,D,E,F,G^	59.6 (4.1)^A,B,C,D^	63.6 (6.0)^A,B,C^	63.7 (2.3)^A,B,C^	57.1 (3.7)^A,B,C,D,E^
ScotchBond Universal (SBU)	40.3 (4.8)^I,J^	42.8 (1.0)^H,I,J^	49.2 (4.6)^E,F,G,H,I^	65.7 (4.2)^A^	64.2 (1.5)^A,B^	61.4 (3.8)^A,B,C^
All-Bond Universal (ABU)	6.9 (2.9)^Q,R^	17.6 (3.0)^N,O,P^	26.0 (1.9)^L,M,N^	37.0 (4.7)^J,K^	51.7 (4.4)^D,E,F,G,H^	40.6 (3.5)^I,J^

^*∗*^Means followed by different capital letters differ statistically by the Tukey test (*p* < 0.001).

## Data Availability

The data used to support the findings of this study are available from the corresponding author upon request.
